# Endometrial Carcinoma Presenting as Vasculitic Sensorimotor Polyneuropathy

**DOI:** 10.1155/2011/968756

**Published:** 2011-07-12

**Authors:** Marketa Vasku, Thomas Papathemelis, Nicolai Maass, Ivo Meinhold-Heerlein, Dirk Bauerschlag

**Affiliations:** Department of Gynecology and Obstetrics, University Medical Center Aachen, Pauwelsstraße 30, 52074 Aachen, Germany

## Abstract

Paraneoplastic syndromes (PNS) are a heterogeneous group of symptoms which are indirectly caused by primary or metastatic tumor. Paraneoplastic polyneuropathy (PNP) is mostly related to small cell lung cancer (5%), prostate, gastric, and breast cancer. Only sporadic cases have been reported to be associated with endometrial cancer. We present a case of a premenopausal woman with severe vasculitic, asymmetric sensorimotor polyneuropathy that developed in conjunction with an endometrial carcinoma responding to surgical therapy of primary tumor combined to steroid therapy. Neurological symptoms such as asymmetrical sensorimotor deficits and painful paresthesias are suspicious when they occur in otherwise healthy women with no medical history. The phenomenon of a paraneoplastic syndrome can point to an underlying malignancy and can be used as marker of progression or regression of the tumor. Due to the rarity of PNP, there is no standard treatment. Recommended therapy is stage-adjusted treatment of the primary tumor.

## 1. Introduction

Paraneoplastic neurologic disorders (PNDs) are remote effects of the cancer affecting any part of the central or peripheral nervous system, presenting with diverse symptoms. Common features of PNDs are usually rapid development, severity of the neurological illness, and appearance before the cancer diagnosis. According to the most widely recognized theory, the genesis is believed to be autoimmune mediated. An autoimmune response of the organism to antigens expressed by tumor cells is assumed. The antigens cause the body's immune system to produce antibodies in an attempt to suppress the cancer. These same antibodies can trigger an autoimmune attack on the brain and the neurological systems [1]. The exact pathophysiological principles are not yet fully understood [2].

The incidence of paraneoplastic polyneuropathy (PNP) fluctuates between 10–40% among all patients with cancer [3], depending on diagnostic criteria. PNP is usually associated with small cell lung carcinoma, prostate, gastric, colon, or breast cancer, and lymphoma [4]. 

We report the particular case of a female patient with progressive vasculitic PNP associated with endometrial cancer presenting as severe asymmetrical sensorimotor deficit resembling a mononeuritis multiplex and proximal motor weakness. 

## 2. Case Report

A 48-year-old, premenopausal Caucasian nulli gravida, was admitted to the Neurological Department of Aachen University Medical Center, complaining of weakness of upper and lower extremities and severe peripheral hypesthesia.

The onset of the patient's symptoms started 3 months before the first admission to the hospital with hypesthesia and pricking paresthesia in upper extremities. The patient gradually developed a severe distal accented loss of muscle power, according to the Medical Research Council Score (MRC) for evaluating peripheral muscle strength 3/5 for upper extremities and 2/5 for lower extremities. All extremities were affected by severe hypesthesia, the pallesthesia was preserved. At the time of the hospital admission she was confined to a wheelchair and unable to attend to own bodily needs without assistance (modified Rankin scale 4/6). The patient's medical history did not reveal any suspicious findings.

Peripheral blood laboratory parameters such as electrolytes, blood clothing, renal and liver function panel, TSH, CRP, homocystein were in normal range except for elevated lipase (68 U/L) and leukocytosis (20,6 G/L). The special extended immunology blood tests were performed. The following targeted parameters have been discovered to be negative: ANA, ds-DNS Ab, ANCA Screening, AMA/LKM-1 Ab Screening, cryoglobulins, and cold agglutinins. The screening for antineuronal antibodies—Anti-Yo,Hu,Ri—was negative. A mild protein elevation 0.59 g/L (normal range: 0.21–0.42 g/L) was found in the cerebrospinal fluid, without evidence of active infection.

The nerve conduction studies (NCSs) showed no sensory nerve action potential in radial and sural nerve; motor NCS in tibial and median nerve demonstrated slightly decreased conduction velocities with decreased amplitude of the compound muscle action potential (CMAP). The electromyelography (EMG) of the anterior tibial muscle did not reveal any spontaneous activity. The results pointed to an asymmetrical miscellaneous sensorimotor polyneuropathy with no evidence of a central motor pathways damage. 

In search of a primary tumor cranial/spine MRI and CT thorax/abdomen were performed without any pathological findings.

**Figure 1:**
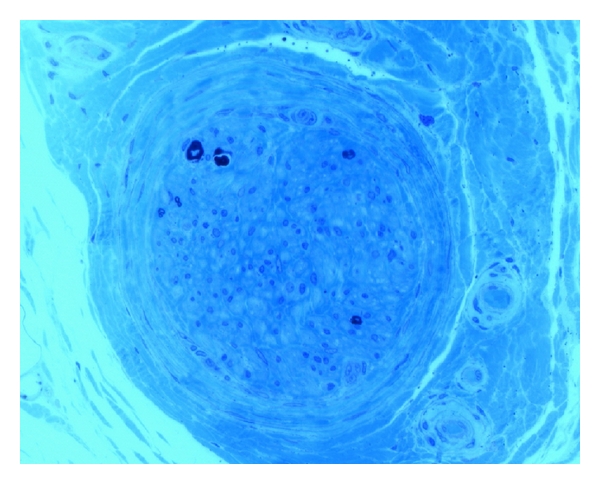
Semithin section of the sural nerve showing a subtotal loss of myelinated nerve fibres. (Semithin toluidine blue section, ×40 oil immersion).

**Figure 2:**
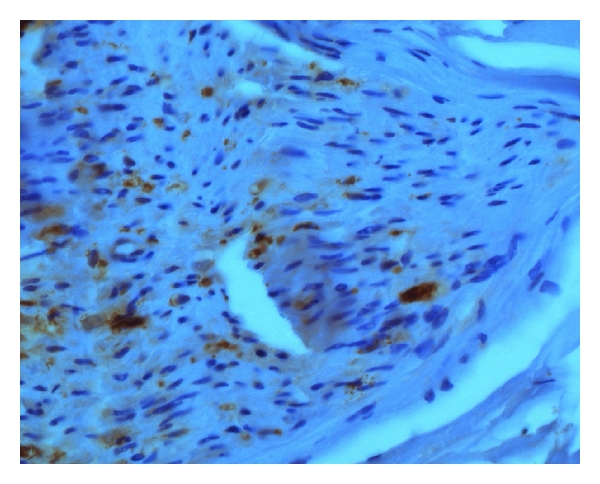
Vasculitis: CD68 immunoreactive macrophages within the epineurium. (Semithin toluidine blue section, ×40 oil immersion).

Sural nerve biopsy revealed an advanced axonal/neuronal neuropathy with a moderate microangiopathy (Figures 1 and 2).

Under the impression of an etiologically unexplained progressive sensorimotor neuropathy corticosteroid therapy (75 mg per day) and symptomatic therapy with pregabalin (150 mg twice a day) was started. 

The gynecological examination because of meno-/metrorrhagia showed an heterogenous and suspicious endometrium. The patient underwent a hysteroscopy with fractional abrasion of the uterus. The histology confirmed a moderately differentiated adenocarcinoma of endometrium, four months after the onset of the first neurological symptoms. An abdominal hysterectomy with bilateral adnexectomy was performed. The postoperative histopathology revealed a G2 endometrioid adenocarcinoma of the uterine corpus with the tumor infiltration less than half the myometrial thickness without cervical or parametrial involvement, but with peritumoral lymphangiosis carcinomatosa. UICC Classification 2010: G2, pT1a, pNx, pMx, L1, R0. FIGO Stage IA. The interdisciplinary tumor board decided on performing the completing systematic pelvic and paraaortic lymphadenectomy. 4 lymph nodes (pelvic/ paraaortic) from 25 lymph nodes were infiltrated. The final classification of the tumor was G2, pT1a, pN1 (4/25), L1, R0 (local). FIGO Stage IIIC2. According to the German interdisciplinary oncologic guidelines an adjuvant radiotherapy combined of paraaortic irradiation with total dose of 45 Gy, parametrial 50, 4 Gy, and vaginal afterloading twice, 8 Gy, was accomplished. 

During the tumor complex treatment an intensive physiotherapy was performed. The duration of the corticosteroid therapy was one month until the hysterectomy with bilateral adnexectomy was performed. To reach better wound healing after the operation prednisolone dose was decreased to 10 mg/die and 7,5 mg/die for 2 months and after all slowly tapered.

The final neurological examination showed an improvement in the motor function in all limbs—Medical Research Council Score (MRC) 4/5 for upper extremities and 3/5 for lower extremities. Furthermore the distal hypesthesia of all extremities persisted. The nerve conduction study verified the clinical finding—almost normal amplitudes and amelioration in velocities in motor tracts. The sensory nerve action potential in lower extremities was still absent.

## 3. Discussion

We introduced a patient with adenocarcinoma of uterus detected through the manifestation of a paraneoplastic neurologic disorder, showing a progressive sensorimotor vasculitic polyneuropathy. The main differential diagnoses of PNP are diabetes, alcoholism, neurotropic infection, metabolic, autoimmune or toxic cause, and traumatic lesion [5]. After excluding other causations of the polyneuropathy [6] in otherwise healthy patient the most reasonable cause was paraneoplastic, although the screening for antionconeural antibodies was negative. Therefore arguments in favor of a remote effect of cancer can only be drawn from indirect criteria. Unfortunately, not all the patient with PNS have identifiable antibodies in their serum [7]. According to Rees [8] the absence of antionconeural antibodies cannot exclude the presence of PNS.

Peripheral neuropathies are among the most common neurologic complications of cancer. PNP is commonly found associated with neoplasms like small cell lung carcinoma, small cell carcinoma of prostate, pancreatic, colon, breast, and gastric cancer. PNP occurs seldom with cancer of female genital organs. Rojas-Marcos et al. [9] reviewed 92 patients with PNSs associated with gynecologic or breast cancer. There was no case of endometrial cancer combined with PNP. Ashour et al. [10] studied 122 reports of gynecologic cancer patients with PNSs and found one case of endometrial cancer presenting as paraneoplastic polyneuropathy with predominantly motor manifestation [11], other cases were related to ovarian cancer [12]. The most successful therapy nowadays is resection of the tumor and/or oncological therapy [13, 14].

## 4. Conclusion

Although the occurrence of PNP and endometrial carcinoma is rare, it can lead to severe clinical signs and may lead to emergency cases. PNP can occur before the neoplastic diseases are detectable, therefore, the prompt recognition of paraneoplastic symptoms facilitates early tumor diagnosis and the timely treatment of tumor and paraneoplastic syndrome. The therapy of PNP itself is not yet standardized because of disease heterogeneity, rarity, and various stages of primary tumor. Corticosteroids, azathioprine, cyclophosphamide, intravenous immune globulin, and other immunomodulatory therapies are tested, but the results are still unsatisfactory. The supplementary symptomatic therapy should be used to alleviate of disabling neurologic symptoms of the patients. Last but not least, immunomodulatory therapy can hypothetically influence tumor growth.
